# The effectiveness of a web-based self-help intervention to reduce suicidal thoughts: A randomized controlled trial

**DOI:** 10.1186/1745-6215-11-25

**Published:** 2010-03-09

**Authors:** Bregje AJ van Spijker, Annemieke van Straten, Ad JFM Kerkhof

**Affiliations:** 1 Department of Clinical Psychology and the EMGO Institute for Health and Care Research, Faculty of Psychology and Education, VU University Amsterdam, Amsterdam, The Netherlands

## Abstract

**Background:**

Suicide, attempted suicide and suicidal thoughts are major public health problems worldwide. Effective face-to-face treatments are Cognitive Behavioural Therapy (CBT), Dialectical Behavioural Therapy (DBT) and Problem Solving Treatment (PST). However, about two-thirds of persons who die by suicide have not been in contact with mental health care services in the preceding year, and many have never been treated. Furthermore, many patients do not disclose their suicidal thoughts to their care provider. This may be out of shame, due to fear of stigma or due to lack of trust in (mental) health care. Since many suicidal individuals seek information online, the internet provides an opportunity to reach suicidal individuals who would not be contacted otherwise. By providing a self-help intervention online, persons can anonymously learn to gain control over their suicidal thoughts. There is convincing evidence that self-help is effective for a number of mental disorders. In this study the effectiveness for suicidal thoughts is examined.

**Methods/Design:**

In this study, a recently developed self-help intervention will be evaluated in a Randomized Controlled Trial. The intervention is based on Cognitive Behavioural Therapy and is aimed at subjects who experience mild to moderate suicidal thoughts. This is defined as a score between 1 and 26 on the Beck Scale for Suicidal Ideation (BSS). Higher and lower scores are excluded. In addition, severely depressed subjects are excluded. In total, 260 subjects will be randomly allocated to the intervention-condition (N = 130) or to the information-control condition (N = 130). Self-report questionnaires will be filled out at baseline, 6 weeks after baseline and 18 weeks after baseline. Primary outcome measure is the reduction in frequency and intensity of suicidal thoughts. Secondary outcome measures are the reduction of hopelessness, anxiety and depression, sleeplessness, worry and quality of life measures.

**Discussion:**

This study is the first to evaluate the effectiveness of a web-based self-help intervention for suicidal thoughts. Several limitations and strengths of the design are discussed.

**Trial Registration:**

Netherlands Trial Register, NTR1689

## Background

Suicide, suicidal behaviour and suicidal thoughts are major public health problems worldwide. In the Netherlands, around 1500 persons die by suicide each year, which accounts for 1% of all deaths [1]. In addition, an estimated 120.000 suicide attempts occur each year [2]. The lifetime prevalence of suicidal thoughts in the adult Dutch population is estimated to be 11% [3]. Suicidal behaviours generally are preceded by suicidal thoughts. A population-based study in the USA showed that 34% of individuals with suicidal thoughts developed specific suicide plans, while 26% directly proceeded to an attempt. In addition, 72% of those who developed a plan proceeded to an attempt [4]. In order to prevent suicidal plans and behaviours, prevention should be aimed at suicidal thoughts.

The majority of persons with suicidal thoughts (80%-82%) meet the criteria of a psychiatric disorder [5]. Most prevalent is depression. Suicidal thinking is one of the most severe symptoms of depression and is characterised by extreme negative thoughts about the self. It has been suggested that relapse in both depression and suicidality is attributed to 'cognitive reactivity' [6,7]. This means that once suicidal thoughts are experienced during an episode of depression, the likelihood that such thoughts will reoccur in a subsequent episode may be increased [8,9]. This 'Differential Activation Theory' of suicidality underlines the importance of aiming prevention at an early stage of the suicidal development.

Many suicidal individuals are reluctant to disclose their suicidal thoughts to others and to seek help. An international review showed that two-thirds were not in contact with mental health care services within the year before the suicide [10]. In the Netherlands, this is estimated to be 64% [11]. An explanation could be that many adults who experience suicidal thoughts do not perceive a need for care [12]. Moreover, there seems to be a negative relationship between levels of suicidal thoughts and help-seeking [13]. Factors that contribute to this 'help-negation effect' may be shame, fear of stigma, lack of trust in mental health care, and prejudices about health care providers. Bearing this in mind, suicidal individuals could benefit from accessible, anonymous help. The internet can play an important role in providing this.

The internet has become an important source of information and means of communication. Worldwide, around 23.5% of the population has access to the internet [14]. Dutch internet access is among the highest in the world (91%) [1]. In general, it has been found that persons with stigmatizing psychiatric illnesses are likely to use the internet to access health information and to communicate with professionals about their condition [15]. The anonymity of the internet may be an important explanation for this [16]. Overall, the internet could be an effective tool in reaching individuals with suicidal thoughts who would not be reached otherwise, or would come into contact with mental health care in a late phase of their suicidal process. For suicidal persons who are being treated, it could be an additional source of help.

Worldwide, there are numerous suicide-related websites. In 2007, a search for English suicide-related websites showed that 13% of the retrieved websites was aimed at prevention or support [17]. An inventory of Dutch suicide-related websites revealed a similar percentage that was aimed at prevention [18]. In general, not much is known about the quality of suicide prevention websites and scientific evaluation of the effectiveness of such websites is lacking. It is therefore not well established what effects prevention websites may have on suicidal individuals.

Although a substantial number of individuals with suicidal thoughts do not seek help, effective treatments exist. Evidence about effectiveness is currently available for Cognitive Behavioural Therapy (CBT), Dialectical Behaviour Therapy (DBT) and Problem Solving Treatment (PST) [19-24]. However, these studies only include face-to-face treatments. In other mental disorders it has been shown that treatments do not necessarily have to be delivered in face-to-face format. Particularly CBT can also be effectively delivered in a self-help format (either a book or through the Internet). Self-help therapy can be defined as a standardized psychological treatment that the patient works through independently at home. Numerous randomized trails have shown that self-help is effective in reducing depressive symptoms, symptoms of anxiety, problem drinking, social phobia and health problems such as headache [25-28]. For suicidal thoughts, a few self-help books are available, but these have not been scientifically evaluated. To our knowledge, no Internet self-help treatments for suicidal thoughts are yet available.

In summary, prevention should be aimed at the onset of suicidal thoughts. The internet can facilitate accessible interventions that may reach suicidal individuals who would not be reached otherwise. In this study, the effectiveness of a recently developed web-based self-help intervention for suicidal thoughts is evaluated in a randomized controlled trial.

## Methods/Design

### Design

This study is a randomized controlled trial comparing a web-based self-help intervention with a waitlist control condition. The intervention is aimed at persons with mild to moderate suicidal thoughts. Subjects in the control condition will have access to an information website and will start the intervention after the experimental group has finished (i.e. after six weeks). Assessments will take place at baseline, 6 weeks after baseline and 18 weeks after baseline.

This study was approved by the Medical Ethics Committee of the VU University Medical Centre (registration number 2008/204).

### Sample size

The sample size of this study is based on the expected effect on the primary outcome measure, i.e. the reduction in frequency and intensity of suicidal thoughts. In order to be able to demonstrate a clinically relevant effect-size of 0.35 with α = 0.05 and β = 0.80, 100 subjects are needed in each condition. When including an expected drop-out of 30%, the total sample size is determined at 260. The effect-size of 0.35 is based on effect-sizes found in other studies into internet interventions, mainly for depression [28,29].

### Inclusion and exclusion criteria

In order to be eligible for the study, subjects have to meet five inclusion criteria. First, the minimum age of subjects is set at 18 years. Second, subjects need to experience mild to moderate suicidal thoughts. This is defined as a score between 1 and 26 on the Beck Scale for Suicide Ideation (BSS) [30]. Subjects with no suicidal thoughts (BSS = 0) are excluded as they can not benefit from this intervention. Subjects with severe suicidal thoughts (BSS ≥ 27) are excluded because they need more extensive care. Third, subjects should not be severely depressed. A severe depression is defined as a score ≥ 40 on the Beck Depression Inventory (BDI) [31]. Severely depressed subjects are excluded because they need more extensive care and may not be focused enough to finish the intervention. Both severely suicidal and severely depressed subjects are advised to seek help elsewhere. Fourth, subjects need to have sufficient command of the Dutch language. Finally, subjects need to have access to internet and an e-mail address.

### Inclusion procedure

In order to reduce attrition, a multi-staged inclusion procedure will be used to filter out impulsive applicants who are likely to drop out. Subjects will be recruited through advertisements in newspapers and via banners on the internet. Through these banners, subjects will be directed to a website where a brief description of the study will be provided. On this website, subjects will also be able to register for the study. To register, subjects are required to fill out their age, the BDI, and the BSS, which will serve to screen for severe depression and suicide risk. Subjects under 18 will receive an automated response that they are too young to participate. Subjects who are severely depressed (BDI ≥ 40) and/or at high risk for suicide (BSS ≥ 27), will receive an automated response which explains that participation is precluded. In addition, the urgent advice to seek help is given, which will be facilitated by providing names and websites of relevant organisations in the Netherlands. An automated response is chosen since subjects have not yet provided us with contact information and informed consent. The BSS also serves to detect absence of suicidal thoughts (BSS < 1). In this case, an automated response explaining that participation is not possible is generated. In this response, subjects are advised to visit the GP since applying for this course might indicate that the subject has other (mental health) problems.

When eligible for participation (BSS < 27 and BDI < 40), subjects are required to fill out an e-mail address. They subsequently will receive an e-mail that contains an information brochure, an informed consent form and a link to the baseline questionnaire. On the consent form, subjects have to fill in their contact information, as well as contact information of their GP. This information will only be used if necessary (see 'safety of subjects'). After giving informed consent and filling in the baseline questionnaire, subjects will be included and randomized.

In addition to filling out an e-mail address, eligible subjects are asked to (voluntarily) fill out additional information on gender, age, educational level, social situation (living alone of together), current use of mental health care, and importance to remain anonymous. This information will be used to compare subjects who are included with those that eventually decline participation. It is expected that a substantial number of potential subjects will decline participation because the intervention is offered in a research context in which it is not possible to remain anonymous. Individuals who prefer to remain anonymous, the potential target population, may differ from persons who participate in the study. By obtaining additional data, differences may be identified. On the website, it will be explained why this information is requested and how it will be processed.

### Randomization

Subjects will be randomized in blocks of 20 after stratification for gender. An independent researcher makes the allocation schedule using random allocation software. Subjects will be informed about the randomization outcome by means of an e-mail, which will also contain a login code for the assigned condition.

### Safety of subjects

Since this study will be conducted in a vulnerable population in which suicide may occur, a safety protocol has been developed. In addition to the pre-, post and follow-up measures, suicidal thoughts will be assessed bi-weekly in both the experimental and the control condition during the first six weeks by means of the BSS and the BDI. The safety protocol will be applied when a subject exceeds the determined cut-off scores, i.e. BSS ≥ 27 or BDI ≥ 40. When this occurs, the researchers will contact the subject by phone on the same day the score is received to assess the suicide risk and contact the GP if necessary. If the phone is not answered, calling will be continued for two days on different times of the day. In case of no response after two days, a standardized e-mail expressing worry will be sent. In this e-mail, it will also be requested to contact the researchers. In addition, it will be explained that the GP will be contacted three days after sending this e-mail. If the GP is not reached, the locum GP is contacted. In case a subject drops out of the study, i.e. does not fill in the questionnaires, an e-mail will be sent with the request to reply and the explanation that the GP will be contacted in case of no reply within three days.

### Intervention

#### Background

Our self-help intervention is based on Cognitive Behavioural Therapy (CBT). CBT nowadays is a widely used and extensively researched treatment. The effectiveness is supported by numerous studies for many mental disorders [32]. In addition to CBT, components of Dialectical Behaviour Therapy (DBT), Problem Solving Treatment (PST), and Mindfulness Based Cognitive Therapy (MBCT) are used in several exercises. DBT is a treatment program developed for borderline personality disorder and has been proven effective in reducing suicidal behaviour [20,22]. PST focuses on improving interpersonal problem solving skills and can be used for the treatment of a variety of mental disorders. It has shown promising results with regard to the treatment of suicidal thoughts [24,33]. MBCT was developed to prevent relapse and recurrence of depression [34], but may also be helpful in the treatment of suicidal thoughts [35]. It combines mindfulness meditation with cognitive therapy.

CBT, DBT, PST and MBCT are all rooted in cognitive therapy [36]. The underlying cognitive model states that emotion and behaviour are influenced by the way events or situations are interpreted. Dysfunctional cognitions can underlie these interpretations and contribute to the development of mental disorders. The cognitive model was originally aimed at explaining psychological processes in depression, but has been expanded to other mental disorders. Although suicidal thoughts and behaviours are not classified as a separate mental disorder, they are related to many mental disorders [37]. Dysfunctional thoughts seen in suicidal individuals often involve hopelessness, helplessness and unlovability [38]. Cognitive therapy techniques aim at restructuring such dysfunctional cognitions. In addition the ruminative, repetitive style of thinking often experienced by suicidal persons can be addressed with cognitive techniques [39-41].

#### Structure

The intervention consists of six modules, each of which can be completed in one week. A module starts by providing information, followed by an assignment and several exercises. Subjects are strongly advised to complete those. In addition, each module contains a number of optional exercises from which the subject can choose the ones that appeal to him. Finally, each module is provided with a number of 'Frequently Asked Questions'. Here, subjects can also actively pose questions which will be answered on a general, non personal level. Consequently no personal feedback or support by a therapist will be offered.

Ideally subjects need to spend at least 15 minutes twice a day to perform the exercises. Every week, an automated e-mail is sent as a reminder of the new module and as motivation to complete it.

#### Content of the modules

- Module 1 aims at teaching subjects to gain more control over their suicidal thoughts. The worrisome, repetitive aspects of suicidal thinking are outlined, and the exercises are aimed at reducing worry.

- Module 2 teaches subjects to recognize and prevent an upcoming crisis. An important message is that seemingly unbearable thoughts can be tolerated. Main goal is that subjects learn to tolerate and regulate intense emotions in a crisis situation.

- Module 3 explains the 'ABC model' which states that emotions (Consequences) are caused by a persons Belief about an Activating event (the trigger). Exercises focus on identifying automatic thoughts.

- Module 4 describes common distortions in thinking. Exercises deal with recognizing and changing them.

- Module 5 is about challenging negative thoughts by evaluating evidence for and against the validity. Exercises also focus on formulating thoughts in a more detailed and neutral way.

- Module 6 explains the possibility of relapse. Also, attention is given to the picture of the future and possible future setbacks. Exercises focus on making a relapse prevention plan and on formulating a more realistic picture of the future.

#### Waiting list control condition

Subjects in the control condition can access a website where information on suicidality is provided. The information is meant to inform them about the nature and possible causes of their thoughts and feelings. Discussed themes are prevalence, risk factors and warning signs. Also, information about treatment in general is provided, as well as links to several mental health organisations. After six weeks, subjects are provided with a login code for the intervention.

### Instruments

Due to the self-help character of this study, all measures are self-reports. No diagnostic instrument is administered.

#### Primary outcome measure

The primary outcome measure in this study is the reduction in frequency and intensity of suicidal thoughts, which is measured by the Beck Scale for Suicide Ideation.

##### Suicidal thoughts

The *Beck Scale for Suicide Ideation*, *self report *(BSS) [42] is a 21-item instrument to detect and measure the severity of suicide ideation. The BSS parallels the *Scale for Suicide Ideation *(SSI), which is a semi-structured interview [43]. Each item is scored from 0 to 2. The first five items of the BSS serve to screen for suicide ideation. If the subject selects zero for both item 4 and 5, he or she is instructed to go to item 20. Otherwise, all items are filled out. The last two items (20 and 21) assess the number of suicide attempts and the intent to die during the last attempt. The total score is obtained by adding the first 19 items, and ranges from 0 to 38. A higher score indicates more severe suicide ideation. The BSS has high internal reliability with Cronbach alpha ranging from 0.87 to 0.97, and moderate test-retest reliability (r = 0.54). The concurrent validity is high with the SSI (0.90 - 0.94), and moderate with the BDI suicide item (0.58 - 0.62).

#### Secondary outcome measures

Secondary outcome measures include depressive symptoms, hopelessness, anxiety symptoms, worrying, quality of life and costs related to health care utilization and production loss.

##### Depressive symptoms

The *Beck Depression Inventory Second Edition *(BDI-II) measures the severity of a depression, and is used to detect the presence of depressive symptoms [31,44]. This questionnaire was introduced in 1961 and is one of the most frequently used and validated instruments to assess depressive symptoms. The total score ranges from 0 to 63 and is obtained by adding the 21 items, which are rated on a 4 point scale (0-3). A higher score indicates more severe depressive symptoms. Interpreting the scores, a total score between 0 and 13 corresponds with minimal depression, 14-19 with mild depression, 20-28 with moderate depression, and 29-63 with severe depression. In both American and Dutch samples, good convergent validity and internal consistency were demonstrated [44].

##### Hopelessness

The *Beck Hopelessness Scale *(BHS) assesses hopelessness [45]. The BHS contains 20 'true-false' statements. Each statement is scored 0 or 1, resulting in a total score range of 0 to 20. A higher score indicates more hopelessness. The BHS is one of the most frequently used measures of hopelessness, and has excellent internal consistency and test-retest reliability. In addition, several studies have supported the predictive validity of the BHS for suicide attempts and suicide [46].

##### Anxiety symptoms

The anxiety subscale of the *Hospital Anxiety and Depression Scale *(HADS-A) is used to assess anxiety symptoms [47]. This subscale consists of 7 items, rated on a 4 point scale (0-3). Total score therefore ranges from 0 to 21, with higher scores indicating more anxiety symptoms. In several normal and clinical Dutch samples, homogeneity and test-retest reliability were good, with Cronbach alpha ranging from 0.80 to 0.84 [48].

##### Worrying

The *Penn State Worry Questionnaire *(PSWQ) was developed in 1990 to measure excessive and uncontrollable worrying [49]. Subsequently, an adapted version was published, which assesses pathological worry in the past week (PSWQ-PW) [50]. The PSWQ-PW is a 15 item self-report inventory with a 7 point rating scale, ranging from 'never' (0) to 'almost always' (6). The total score ranges from 0 to 90, with a higher score indicating more worrying. The PSWQ-PW shows good reliability and substantial convergent validity. It assesses both weekly status of worry and treatment-related changes of worry during treatment, which makes it suitable for monitoring pathological worry in research settings [50].

##### Quality of life

The *EuroQol *(EQ-5D) [51] is a standardised non disease specific instrument for describing and valuing health related quality of life. It consists of five items (mobility, self-care, usual activities, pain/discomfort and anxiety/depression). Each item is rated as 'no problem' (1), 'some problem' (2), or 'extreme problem' (3). The resulting health state can therefore be expressed by a five-digit number. In addition, it is required to rate current health state on a thermometer ranging from 0 (worst imaginable health state) to 100 (best imaginable health state).

##### Costs: health care utilization and production loss

Health care utilization and production loss are evaluated by means of the *Trimbos/iMTA questionnaire for Costs associated with Psychiatric Illness *(TiC-P) [52]. The TiC-P consists of two different parts that can be administered separately. Part I is concerned with measuring the direct costs of care consumption of subjects with psychiatric disorders. It consists of 15 items, but can be customized for the study population. Items that are considered irrelevant can be left out. Part II is meant to determine indirect costs resulting from production loss associated with psychiatric disorders. It consists of the *Short Form Health and Labor Questionnaire *(SF-HLQ) [53]. The SF-HLQ contains three modules covering absence from paid employment, production loss without absence from paid employment and impediments to paid of unpaid employment.

##### Functioning

The *Work and Social Adjustment Scale *(WSAS) [54] measures impaired functioning attributable to an identified problem. It is a five item scale, with each item being rated on a 0 (no impairment at all) to 8 (very severe impairment) scale. The WSAS is valid and reliable. Internal consistency ranges from 0.70 to 0.94. Test-retest correlation was 0.73.

### Statistical analyses

To test the hypothesis that the self-help intervention is superior to the control condition, the analysis will be conducted on an Intention to Treat basis following the pertinent BMJ & Consort guidelines. Missing observations at follow up will be imputed by regression imputation or multiple imputations, stratified for predictors of outcome and loss to follow-up. Relative improvements in frequency and intensity of suicidal thoughts in the experimental group in comparison with the control group will be calculated by Cohen's *d*. For this confirmatory analysis the primary outcome measure is used (BSS). To the exploratory analyses of the secondary outcome measures, a Bonferroni correction will be applied to control the overall Type I error rate.

## Discussion

This paper describes the study protocol of a randomized controlled trial comparing a web-based self-help intervention to reduce suicidal thoughts with a control condition. To our knowledge, this is the first self-help application targeted specifically at suicidal thoughts. This aspect simultaneously might give rise to the idea that focusing mainly on suicidal thoughts could instigate suicidal thoughts or even suicidal behaviour. Even though this is a widespread belief, this idea is not validated in psychology or psychiatry [55].

The described study design has several methodological limitations. First, internet interventions in general and self-help applications in particular are subjected to high attrition rates [56]. Dropout from the study (dropout attrition) can introduce a selection bias and therefore be a threat to validity. In order to reduce dropout attrition, the questionnaires are presented separately from the intervention website and reminders are sent. Dropout from the intervention (nonusage attrition) may result in an underestimation of the effect size. In order to reduce nonusage attrition, subjects receive a motivating (automated) e-mail each week. Besides trying to reduce attrition, it will be analysed thoroughly (as has been suggested by Eysenbach) [56].

Second, anonymous participation is not possible because subjects are at risk for suicide, and we wanted to be able to monitor them and take action if necessary. As a result, the study population may differ from the target population. In order to identify possible differences, several demographics are obtained before registration.

Finally, no diagnostic interview is conducted. This makes it impossible to diagnose psychiatric disorders, but is in keeping with the self-help character of the intervention. It is also in accordance with the eventual purpose of use, since the intervention will become available for people with suicidal thoughts in general, irrespective of the presence of a possible psychiatric disorder. A feature of the described design that decreases the generalizability to this target group, is that severe suicidal and severe depressed subjects are excluded. The rationale for this is that they need more extensive care.

One of the strengths of this study lies in the design itself since the number of randomized controlled trails concerning suicidality is relatively limited [23,57]. In general, suicide intervention research has been described as challenging due to methodological difficulties and ethical considerations [58]. As a result, there still is a paucity of information on the effectiveness of treatments for suicidal persons. The present study can contribute to increasing knowledge on this subject. A second and related unique aspect of this study is that suicidal thoughts are the primary target of the intervention. Often, suicidal thoughts are treated as part of a depression. However, suicidal thoughts not only occur in depression, but are related to many mental disorders.

## Competing interests

The authors declare that they have no competing interests.

## Authors' contributions

AK obtained funding for this study. All authors contributed to the design of this study and the development of the intervention. BS drafted the manuscript and will carry out the study. All authors contributed to further writing of the manuscript and read and approved the final manuscript.
